# Dr. Lucien E. Ellis

**Published:** 1913-09

**Authors:** 


Dr. Lucien E. Ellis, Buffalo, 1879, died at his home in Detroit July 12, aged 63. He had been a member of the School Board for 14 years.

